# *CsWAK12*, a novel cell wall-associated receptor kinase gene from *Camellia sinensis*, promotes growth but reduces cold tolerance in *Arabidopsis*

**DOI:** 10.3389/fpls.2024.1420431

**Published:** 2024-11-28

**Authors:** Qiong Wu, Xiaoyu Jiao, Dandan Liu, Minghui Sun, Wei Tong, Xu Ruan, Leigang Wang, Yong Ding, Zhengzhu Zhang, Wenjie Wang, Enhua Xia

**Affiliations:** ^1^ Tea Research Institute, Anhui Academy of Agricultural Sciences, Hefei, Anhui, China; ^2^ State Key Laboratory of Tea Plant Biology and Utilization, Anhui Agricultural University, Hefei, China

**Keywords:** *Camellia sinensis*, wall-associated kinases (WAKs), plant growth, CBF, cold stress

## Abstract

Cold significantly impacts the growth and development of tea plants, thereby affecting their economic value. Receptor-like kinases (RLKs) are thought to play a pivotal role in signaling the plant's response to cold and regulating cold tolerance. Among the RLK subfamilies, wall-associated receptor-like kinases (WAKs) have been investigated across various plant species and have been shown to regulate cell growth and stress responses. However, the function of WAK genes in response to cold stress in tea has yet to be studied. In a previous investigation, we identified the *WAK* gene family from Camellia sinensis and isolated a specific *WAK* gene, *CsWAK12*, which is induced by abiotic stresses. Here, we demonstrate that CsWAK12 is involved in the regulation of cold tolerance in tea plants. CsWAK12 was rapidly induced by cold, peaking at 3 hours after treatment at 4°C (10-fold increase). Heterologous overexpression of *CsWAK12* (35S:*CsWAK12*) in *Arabidopsis* promoted plant growth by enhancing root length and seed size under normal conditions, although it reduced cold resistance compared to the wild type. Under cold stress, the transgenic plants exhibited a lower survival rate and significantly altered levels of superoxide dismutase (SOD) activity and malondialdehyde (MDA) content compared to the wild type (WT). Furthermore, the expression of C-repeat/dehydration-responsive element binding factor (CBF) genes was diminished in CsWAK12-overexpressing transgenic Arabidopsis plants following cold treatment. Transcriptome analysis revealed that genes associated with the CBF pathway, such as transcription factor genes (ERF53, ERF54, and DREB2A) were markedly reduced in the overexpression line. These data suggest that CsWAK12 acts as a negative regulator, reducing the cold tolerance of transgenic Arabidopsis by mediating the CBF pathway. Therefore, CsWAK12 may serve as a candidate gene for the molecular breeding of cold resistance in tea plants.

## 1 Introduction

Wall-associated kinases (WAKs) constitute a distinct subfamily of receptor-like kinases (RLKs). Their extracellular domains are tightly bound to the pectin of the cell wall and play a crucial role in the plant’s response to stress (He et al., 1996; Anderson et al., 2001). In recent years, the *WAK* gene family has been identified in various plants, including *Arabidopsis*, tobacco, cotton, tomato, rice, and barley, and has been confirmed to be involved in plant growth and development, pathogen response, abiotic stress response, and other physiological processes (Dou et al., 2021; Zhang et al., 2005; Tripathi et al., 2020; He et al., 1999; Kurt et al., 2020; Yu et al., 2022). Antisense *WAK2* or *WAK4* in *Arabidopsis*, which resulted in a 50% decrease in WAK protein levels, led to reduced cell elongation, blocked lateral root development, and dwarfed plants (Lally et al., 2001; He et al., 1999). *OsWAK10* in temperate *Oryza japonica* accessions regulates cellulose synthesis in the secondary cell wall by sensing pectic cell wall components, thereby controlling plant height and stem strength in rice (Cai et al., 2023). The cotton gene *GhWAK7A* mediates chitin-induced signaling pathways, activating downstream gene expression by phosphorylating lysin-motif-containing receptor-like kinases (LYKs/CERK1), specifically GhLYK5 and modulating the chitin-induced association between GhLYK5-GhCERK1 to enhance resistance against the infections of *Verticillium dahliae* and *Fusarium oxysporum f.* sp. *vasinfectum* (Lin et al., 2020). The overexpression of *OsWAK112* in rice and *Arabidopsis* significantly decreased plant survival under salt stress, while the knockdown of *OsWAK112* in rice enhanced plant survival under the same condition. This indicates that *OsWAK112* negatively regulates plant salt responses by inhibiting ethylene production, possibly through direct binding with OsSAMS1/2/3 (Lin et al., 2021). Additionally, silencing *CaWAKL20* improved the heat tolerance of pepper; however, *Arabidopsis CaWAKL20*-OE lines exhibited decreased sensitivity to abscisic acid (ABA), resulting in increased heat sensitivity. This suggests that *CaWAKL20* negatively modulates plant thermotolerance by reducing the expression of ABA-responsive genes (Wang et al., 2019).

Tea [*Camellia sinensis* (L.) O. Ktze] is a traditional plant renowned for producing a popular beverage in China, celebrated for its refreshing qualities, pleasant aroma, and lively palate. With the rise of new-style tea consumption, the value of tea plantations in China has increased significantly. However, temperature variations associated with warm and cold climates pose limitations on the cultivation and growth of the tea plant, which is typically classified as a tropical and subtropical crop. Low temperatures can cause the freezing of cell membranes, leading to cell death, leaf damage, and restricted growth. In recent years, significant advancements have been made in identifying cold stress-responsive RLKs and their associated signaling networks. For instance, *Arabidopsis* PHLOEM INTER-CALATED WITH XYLEM-LIKE 1 (AtPXL1) is induced by cold and heat stress, but not by drought (Jung et al., 2015). The overexpression of the brassinosteroid receptor (BRI1) gene *AtBRI1* resulted in a higher germination frequency of *Arabidopsis* seeds under cold stratification, while the mutant exhibited reduced sensitivity to cold stratification (Kim et al., 2019). However, the roles of WAKs in response of tea plant to cold stress have not yet been reported.

In our previous research, we identified the tea *WAK* gene family through *in silico* analysis and examined its expression patterns using a microarray dataset (Jiao et al., 2023). In this study, we isolated a previously identified *WAK* gene, designated *CsWAK12* (CSS0006198), which exhibited significant cold sensitivity in tea plants. We further characterized the *CsWAK12* gene to assess its expression at a chilling temperature of 4°C and investigated plants that heterogeneously overexpressed this gene when subjected to sub-zero temperatures of −8°C. The CsWAK12 protein was localized to the membrane. Additionally, the ectopic expression of *CsWAK12* in *Arabidopsis* resulted in a notable decrease in cold tolerance in the transgenic plants. Specifically, superoxide dismutase (SOD) activity was reduced, and malondialdehyde (MDA) content was inhibited in the *CsWAK12* transgenic lines in *Arabidopsis*. Furthermore, quantitative real-time PCR and transcriptome analysis revealed that *AtCBFs* were significantly influenced by *CsWAK12*.

## 2 Materials and methods

### 2.1 Plant materials and stress treatment

The tea cultivar “Shu Cha Zao” (SCZ) was cultivated in the Experimental Tea Garden of the Tea Research Institute at the Anhui Academy of Agricultural Sciences, located in Huangshan, Anhui (118.26E, 29.69N). To analyze the tissue expression pattern, apical buds, young leaves, stems, roots, and flowers were collected from the same SCZ plant in the garden in its natural state. Tender branches, approximately 15–20 cm in length, were collected from SCZ and inserted into water-saturated flower mud for cold treatment in a 4°C culture chamber. Conditions in the chamber were maintained at 60% relative humidity, with a 12-h photoperiod and a light intensity of 2,000 lux. Control samples were placed in a tissue culture room at 28°C, also under 60% relative humidity and a 12-h photoperiod with the same light intensity. Young leaves were collected after 0, 3, 6, 12, 24, and 48 h of each treatment. All samples were processed in the morning at 10:00 a.m., wrapped in foil, immediately frozen in liquid nitrogen, and stored at −80°C.

### 2.2 RNA isolation, cDNA synthesis, and quantitative real-time PCR analysis

Total RNA was extracted from plant materials using the RNAprep Pure Plant Plus Kit (Tiangen, Beijing, China) and subsequently subjected to reverse transcription using PrimeScript™ RT Master Mix (TAKARA, Tokyo, Japan), following the manufacturer’s instructions. Quantitative reverse transcription polymerase chain reaction (qRT-PCR) was conducted on a LightCycler^®^ 96 Instrument (Roche, California, USA) utilizing SYBR^®^ Green Pro Taq HS Premix (AGBIO, Hunan, China) by a three-step PCR reaction procedure. The reaction volume was set to 20 μL, and the thermal cycling program consisted of an initial denaturation step at 95°C for 30 s, followed by 40 cycles of denaturation at 95°C for 5 s and annealing/extension at 60°C for 30 s. Each sample was technically replicated three times and the relative expression levels were normalized to the *CsACTIN* gene (Shen et al., 2019). The primers used for qRT-PCR are detailed in **Supplementary Table S1A**.

### 2.3 Plant transformation and generation of overexpressing *Arabidopsis* plants

The complete coding sequence of *CsWAK12* was ligated into the binary vector pCAMBIA3301 (Pyeast, Shaanxi, China), which contains EcoRI and BamHI restriction sites and is regulated by the CaMV35S promoter, utilizing specific primers (**Supplementary Table S3B**). The relative expression levels were normalized to those of the *AtUBQ10* gene. The recombinant plasmid was introduced into the *Agrobacterium tumefaciens* strain GV3101 (Tsingke, Beijing, China) using the freeze–thaw method. A transgenic *Arabidopsis* strain was generated by transforming wild-type (WT) *Arabidopsis* (Columbia-0) through the floral dip method. T1 plants were screened on 1/2 Murashige-Skoog (MS) Modified Plant Media containing 0.05% glufosinate after sterilization with 20% NaClO and 75% alcohol and were confirmed via PCR. T3 transgenic progeny were selfed from T1 for at least two generations and verified by sequencing (Tsingke, Beijing, China).

In brief, plants were grown in a controlled growth chamber under a 16-h light and 8-h dark cycle, maintained at temperatures of 23/22°C and 60% relative humidity for optimal growth. T3 transgenic and WT Arabidopsis seeds were sown on the surface of the aforementioned 1/2 MS Media. The total root growth of 10-day-old seedlings was measured using vernier calipers. Ten seedlings were taken from each strain, with three replicates for each. To compare the seed length of various Arabidopsis types, seeds harvested at the same time were measured and recorded using ImageJ (Schneider et al., 2012). A minimum of 30 seeds from each line were measured.

### 2.4 Evaluation of cold tolerance in transgenic *Arabidopsis* seedlings

Following the previously reported protocol, the freezing assay was conducted with minor modifications (Xie et al., 2018; Yang et al., 2023). To evaluate the survival rates of different types of *Arabidopsis* types under cold stress and tolerance, seedlings were cultivated on medium under normal conditions for 2 weeks before exposure to −8°C for 2 h. Subsequently, all seedlings were kept in the dark at 4°C for at least 12 h post-chilling and then returned to normal conditions for 6 days. After the recovery period, the presence of new young leaves was considered indicators of viable seedlings.

To assess the phenotypic changes in different *Arabidopsis* types under cold stress, 10-day-old seedlings were transferred to nutrient soil composed of vermiculite and perlite in a 2:1:1 ratio. These 3-week-old seedlings were then subjected to 4°C for 1 week for cold acclimation, followed by exposure to −15°C for 1.5 h, hereinafter referred to as CA. The 4-week-old seedlings in soil were divided into three groups: one group was treated at the low temperature of −8°C for 2 h (NA1), another group was treated at −15°C for 1 h (NA2), and the remaining group was maintained under normal conditions as a control.

To investigate the physiological changes in different *Arabidopsis* types, 4-week-old seedlings under normal conditions were transferred to −6°C for 0, 1.5, 3, and 6 h to measure SOD activity and MDA content. SOD activity was measured using the SOD Activity Assay Kit (WST-1 Method), while MDA content was assessed using the MDA Content Assay Kit (Solarbio, Beijing, China) to evaluate the cold tolerance of transgenic *Arabidopsis* seedlings over various durations.

### 2.5 RNA-seq analyses of cold−related genes in transgenic *Arabidopsis* seedlings

To investigate the mechanistic network of *CsWAK12* in response to cold, comparative transcriptomic analyses were conducted using 4-week-old seedlings from transgenic *Arabidopsis* lines and WT plants. These seedlings were subjected to −6°C for 3 h, with normal conditions serving as the control. Total RNA was extracted from both overexpressing *Arabidopsis* and WT plants using the Total RNA Extractor (Trizol) and quantified with the Qubit^®^ 2.0 Fluorometer (Invitrogen, CA, USA) employing Qubit™ RNA High Sensitivity Kits (Invitrogen, CA, USA) for transcriptome analyses. Sequencing was performed on the Illumina Hiseq X Ten platform, and the resulting reads were mapped to the reference genome of *A. thaliana* (TAIR10) using HISAT2 (Kim et al., 2015). Transcript expression was assessed using RSeQC, and transcript abundance was estimated in transcripts per million (TPM) (Wang et al., 2012). Differentially expressed genes (DEGs) were analyzed with DESeq2 and selected based on Student’s *t*-test, applying a significance threshold of *p* < 0.05 and a fold change (FC) greater than 2. A Gene Ontology (GO) enrichment analysis was performed using topGO (Alexa et al., 2006). Kyoto Encyclopedia of Genes and Genomes (KEGG) pathway enrichment analysis of DEGs was conducted using the R package clusterProfiler (Yu et al., 2012).

C-repeat/dehydration-responsive element binding factor (CBF) genes, including *AtCBF1* (*AT4G25490*), *AtCBF2* (*AT4G25470*), and *AtCBF3* (*AT4G25480*), were quantified by qRT-PCR. Additionally, the expression level of *CsWAK12* in transgenic lines was measured. *Arabidopsis AtUBQ10* (*AT4G05320*) served as the reference gene in qRT-PCR (Liu et al., 2022). The primer sequences for the genes utilized are listed in **Supplementary Table S3E**. Relative gene expression values were calculated using the 2^−ΔΔCt^ method.

### 2.6 Statistical analysis

All experiments were conducted with a minimum of three biological replicates. Statistical analyses were performed using Microsoft Excel (Microsoft, Washington, USA) and IBM SPSS Statistics 20 (IBM, New York, USA). The data are presented as mean ± standard deviation (SD). Different letters indicate significant differences at *p* < 0.05, while the symbol ** denotes significant differences at *p* < 0.01, as determined by one-way ANOVA.

## 3 Results

### 3.1 Molecular characteristics of *CsWAK12* and homoeologous genes

The *CsWAK12* open reading frame (ORF) was isolated from the tea variety SCZ based on the available putative sequence information in the TPIA database (Gao et al., 2023). Our previous study indicated that *CsWAK12* is located on chromosome 4 of the tea plant (Xiao-Yu et al., 2023). The isolated full-length mRNA of *CsWAK12* (accession no. PP739783) consists of 2,265 nucleotides (nt) that encode a protein of 754 amino acids with an estimated molecular mass of 83.39 kDa and an isoelectric point of 5.70.

**Figure 1:**
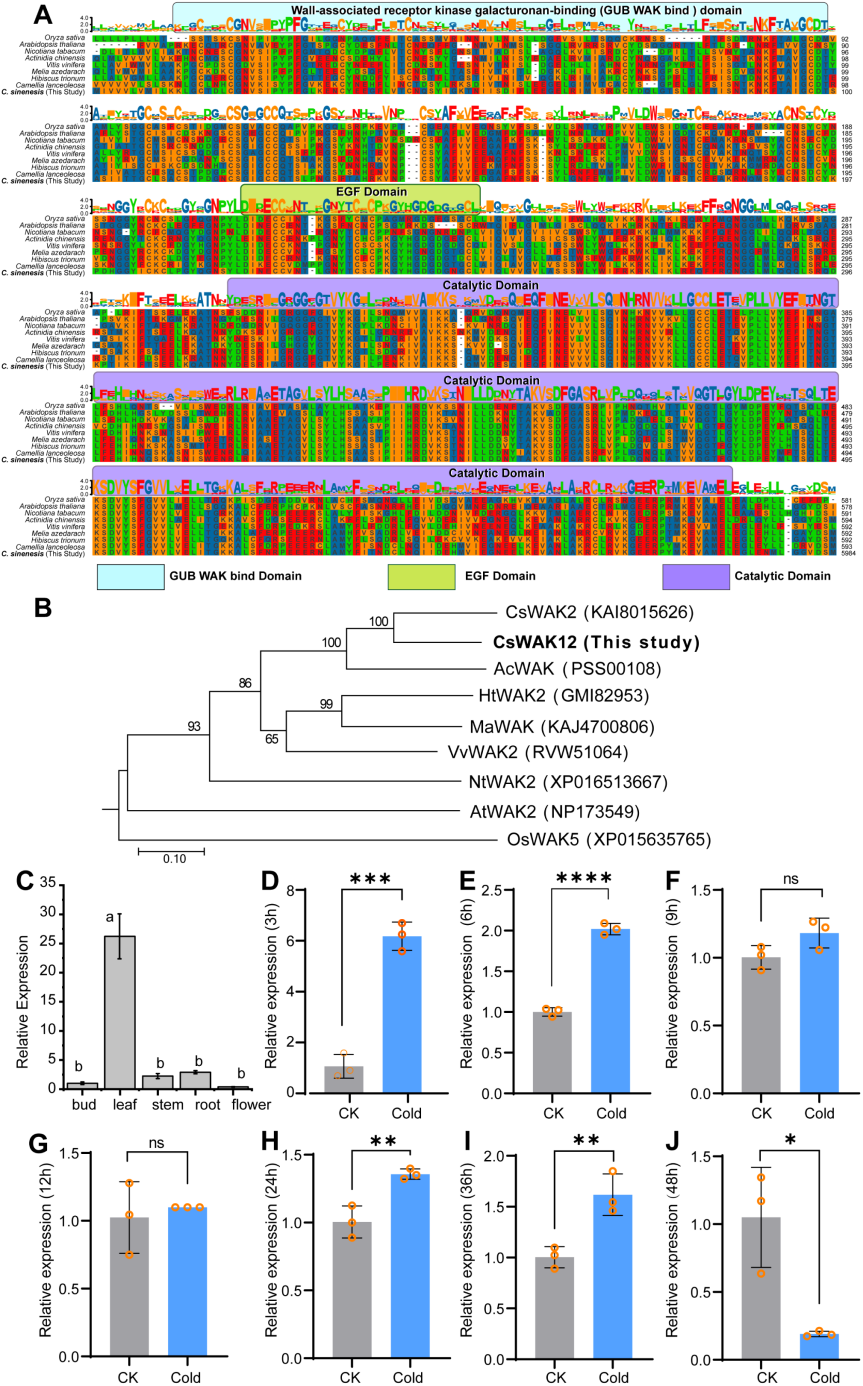
Sequence alignment, phylogenetic analysis, and expression pattern of *CsWAK12*. **(A)** Multiple sequence alignment of homologous genes of *CsWAK12.* Different domains are represented by different colors, where the GUB WAK bind domain is light blue, the EGF domain is green, and the catalytic domain is purple. **(B)** The phylogenic tree of *CsWAK12* in different plant species was plotted by the MegAlign software. **(C)** qRT-PCR analysis of the *CsWAK12* transcript in different tissues of SCZ. The total RNA was isolated from the samples of bud, leaf, stem, roots, and flowers. **(D–J)** The expression levels of *CsWAK12* in tea leaves under low-temperature treatment. Error bars represent ± SD (*n* = 3). Different letters indicate significant differences at *p <* 0.05 according to two-way ANOVA (Duncan’s multiple range test). Statistically significant differences were indicated by *p < 0.05; **p < 0.01; ***p < 0.001;****p < 0.0001 according to unpaired *t*-test.

The protein BLAST analysis of the complete amino acid sequences revealed the presence of at least three domains in the CsWAK12 protein, including the wall-associated receptor kinase galacturonan-binding domain, the epidermal growth factor-like domain, and the catalytic domain (**Figure 1A**). This finding is consistent with homologous genes from *Arabidopsis thaliana* (NP173549.1), *Actinidia chinensis* (PSS00108.1), *Camellia lanceoleosa* (KAI8015626.1), *Hibiscus trionum* (GMI82953.1), *Melia azedarach* (KAJ4700806.1), *Nicotiana tabacum* (XP016513667.1), *Oryza sativa* (XP015635765.1), and *Vitis vinifera* (RVW51064.1). These results suggest that the structure of *CsWAK12* and homologous genes is relatively conserved, implying that they may perform similar functions. Furthermore, the phylogenetic analysis indicated that the CsWAK12 protein shares greater similarities with CsWAK2 from *Camellia lanceoleosa* (**Figure 1B**).

### 3.2 Expression patterns of *CsWAK12* in tea plant

To investigate the potential functions of *CsWAK12*, we examined its relative expression across various tissues of naturally growing tea plants. We performed qRT-PCR to analyze the expression patterns of *CsWAK12* in different tissues of SCZ, including bud, leaf, stem, root, and flower. The results indicated that the *CsWAK12* gene exhibited the highest expression in young leaves and the lowest expression in apical buds. Notably, *CsWAK12* was expressed in all examined organs, with the highest levels found in leaves, and the lowest in flowers, suggesting that *CsWAK12* plays significant roles in leaf development (**Figure 1C**).

To further explore the potential function of *CsWAK12* in the cold response of tea, we subjected long fresh shoots of SCZ to low-temperature conditions (4°C) for indoor simulated cold stress. qRT-PCR analysis revealed that *CsWAK12* was upregulated under cold stress, exhibiting more than a twofold increase in relative expression values. Notably, *CsWAK12* transcripts peaked at 3 h at 4°C, reaching approximately a 10-fold increase compared to the control treatment (**Figure 1D**). This increased expression was also significantly greater at 6, 24, and 36 h post-treatment (**Figures 1E–I**), while a significant decrease at 48 hours (**Figure 1J**).

### 3.3 Overexpression of *CsWAK12* improves the growth of *Arabidopsis*

**Figure 2:**
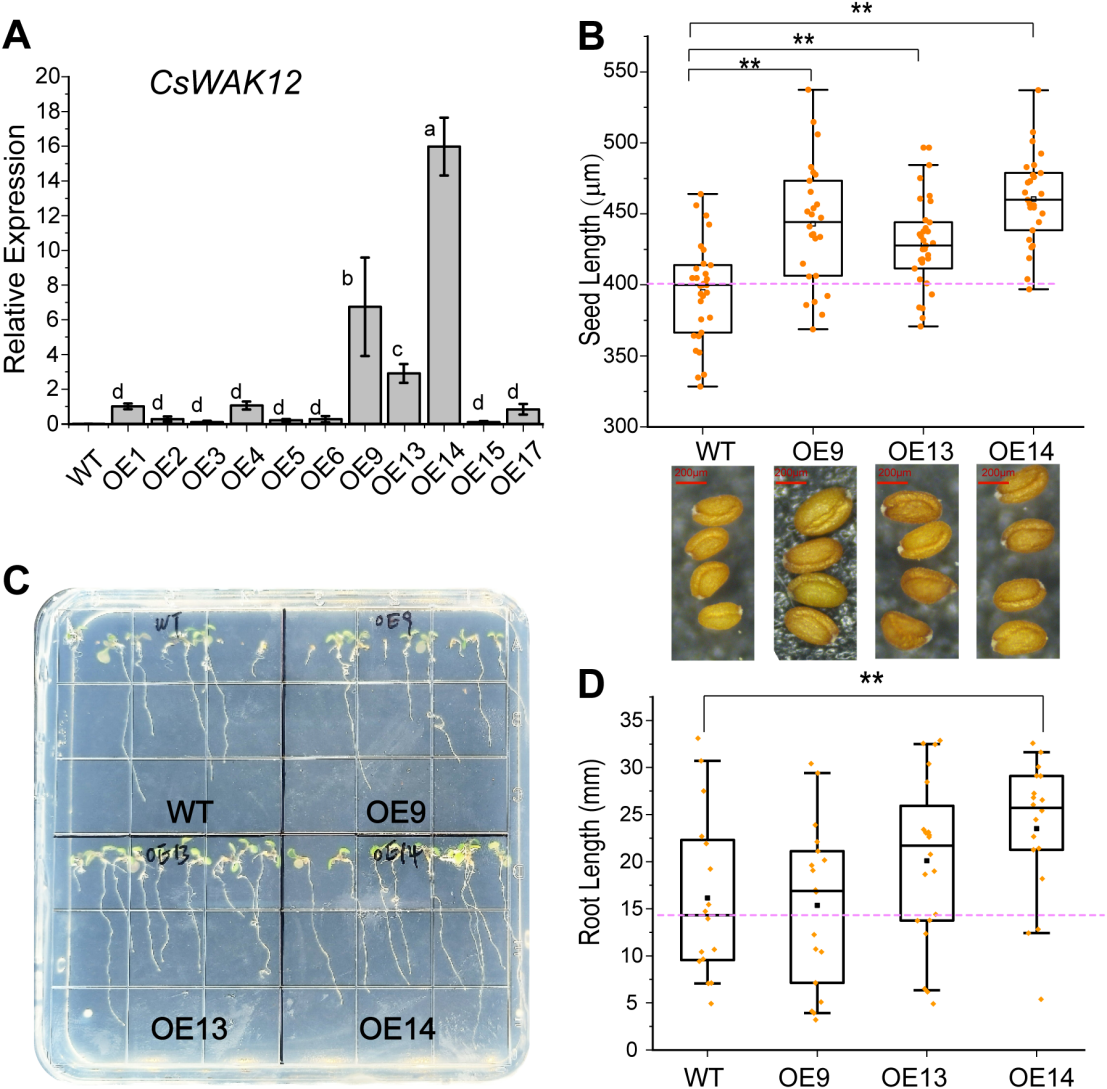
Overexpression of *CsWAK12* promotes the growth in transgenic *Arabidopsis*. **(A)** The transcription level of the *CsWAK12* gene in different lines. **(B)** The boxplot showed the seed size of transgenic lines. The lower graph showed the seed of transgenic lines. **(C)** The phenotypes of *CsWAK12* transgenic lines grown on medium. **(D)**The boxplot showed the root length of transgenic lines. The error bars indicate the SDs from three biological replicates. ** indicates a significant difference at the 0.01 level. Different lowercase letters indicate significant difference (P–0.05).

The *CsWAK12* gene, driven by the CaMV35S promoter, was successfully transformed into *Arabidopsis*. Eleven overexpression lines of *CsWAK12* were obtained from which three lines exhibiting notably high expression levels were selected: OE-*CsWAK12*#9, OE-*CsWAK12*#13, and OE-*CsWAK12*#14, referred to as OE9, OE13, and OE14, respectively (**Figure 2A**). These lines were chosen from T3 homozygous lines for further characterization. Phenotypic analysis of the transgenic lines indicated that *CsWAK12* promotes growth in *Arabidopsis*, as evidenced by increased root length and seed size (**Figure 2B**). Notably, the average root length of OE14 was 7.34 mm longer than that of the WT. The average seed lengths measured 460.51 μm for OE14, 442.98 μm for OE9, and 428.06 μm for OE13, while the WT measured 395.65 μm (**Figures 2C, D**).

### 3.4 Overexpression of *CsWAK12* reduces the cold tolerance of *Arabidopsis*

**Figure 3:**
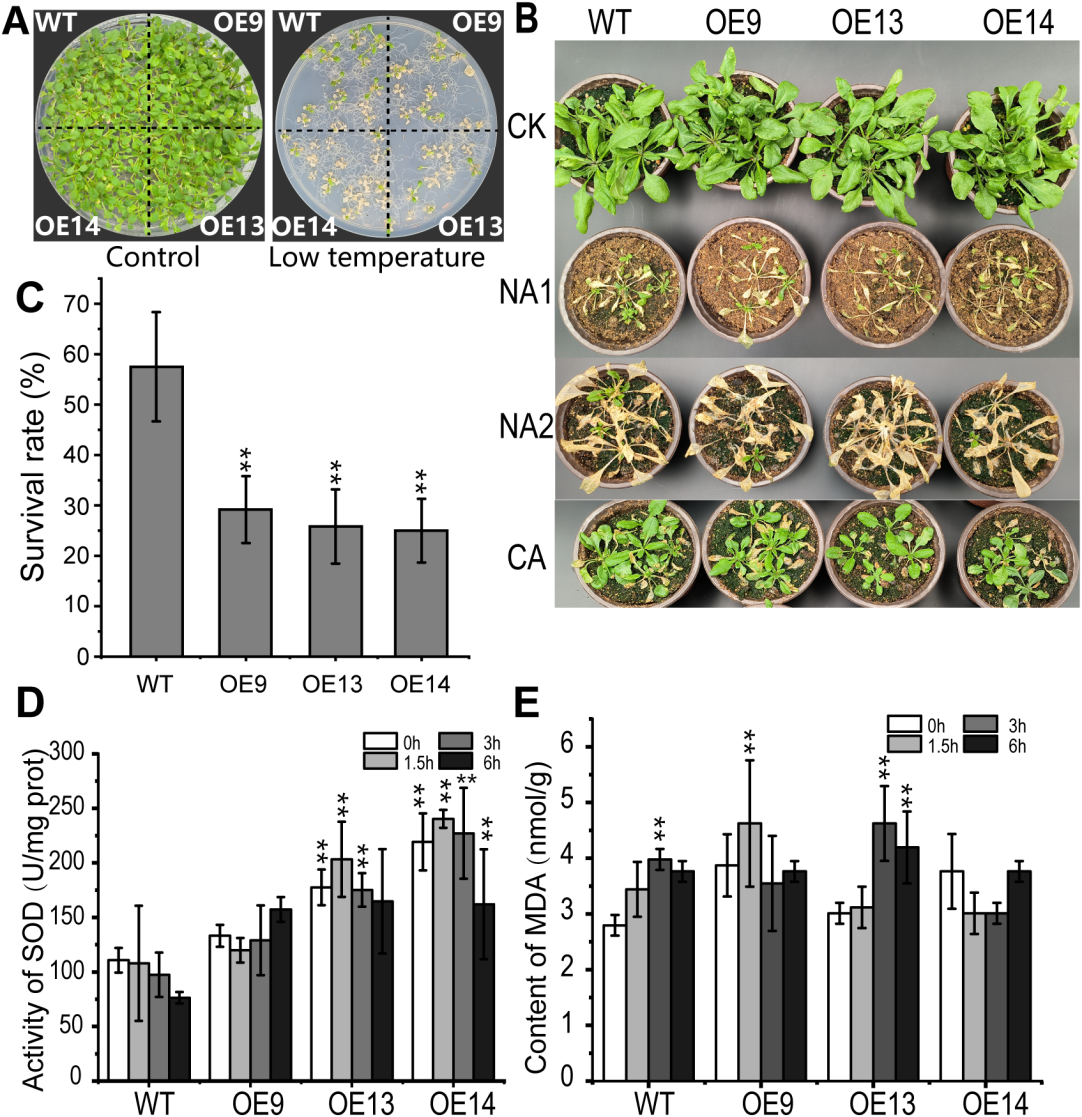
Overexpression of *CsWAK12* in *Arabidopsis* reduced cold tolerance. **(A)** The left panel shows the phenotypes of wild-type and transgenic plants growing normally for 2 weeks, while the right panel illustrates the phenotypes of wild-type and transgenic plants after cold treatments at −8°C for 2 (h) **(B)** Phenotypes of the transgenic lines and WT plants after freezing. Four-week-old pot-grown *Arabidopsis* plants were treated with −8°C for 2 h (NA1) and −15°C for 1 h (NA2), after which they were recovered for 7 days. **(C)** Survival rate of WT and transgenic Arabidopsis lines under cold stress. **(D)** Activity of SOD and **(E)** content of MDA of the transgenic lines and WT plants after −6°C. The error bars indicate the SDs from three biological replicates. ** indicates a significant difference at the 0.01 level.

To assess the cold tolerance of *CsWAK12* transgenic lines, 2-week-old WT and transgenic seedlings grown on 1/2 MS plates were transferred to −15°C for 1 h (**Figure 3A**). Additionally, 4-week-old WT and transgenic seedlings grown in nutrient soil were subjected to −8°C for 2 h and −15°C for 1 h. We observed that the overexpression of *CsWAK12* resulted in an altered cold tolerance phenotype (**Figure 3B**). All three transgenic lines exhibited reduced cold tolerance, as evidenced by lower survival rates. While the survival rates of WT and transgenic lines were compared under normal growth conditions, they differed significantly following cold treatment. Specifically, 59% of WT *Arabidopsis* survived exposure to −8°C for 2 h, whereas only 27% of transgenic *Arabidopsis* survived (**Figure 3C**). In summary, the survival rate of transgenic plants without cold acclimation was significantly lower than that of WT plants. These transgenic plants exhibited stunted growth, shorter root lengths, reduced plant height, and wilted, yellowing rosette leaves after exposure to freezing conditions. Although cold-acclimated transgenic plants demonstrated some degree of cold resistance, their growth rate remained slower, and their survival rate was still diminished. Furthermore, transgenic *Arabidopsis* demonstrated significantly altered levels of SOD activity and MDA (**Figures 3D, E**). The activity of SOD in WT plants gradually decreased with prolonged cold treatment while the transgenic lines exhibited an overall increase in activity. In contrast, the MDA content in WT plants increased steadily with extended cold exposure. In the transgenic line OE9, MDA levels rose rapidly within the first 3 h but subsequently decreased. The transgenic line OE13 exhibited a similar trend to the WT, albeit with a higher growth rate. Additionally, MDA levels in line OE14 displayed an inhibition pattern after exposure to cold (**Supplementary Table S4A**).

### 3.5 *CsWAK12* affects the expression of cold-responsive genes in *Arabidopsis*

**Figure 4:**
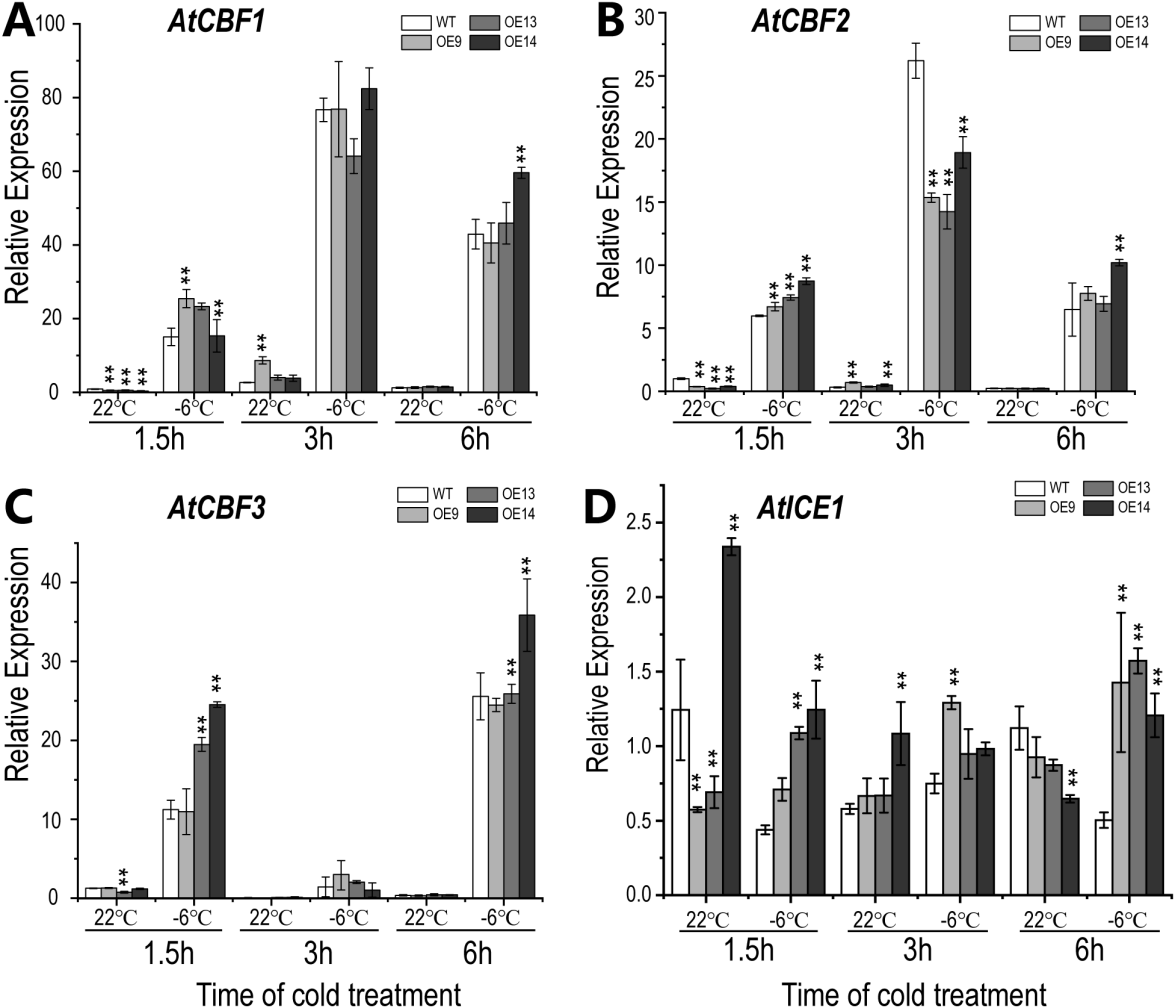
Expression pattern of *CsWAK12* and cold-related genes in *Arabidopsis* plants. The expression of *AtCBF1* **(A)**, *AtCBF2* **(B)**, *AtCBF3* **(C)**, and *AtICE1* **(D)** in transgenic lines and WT. The error bars indicate the SDs from three biological replicates. ** indicates a significant difference compared with WT under each treatment at the 0.01 level.

The signal transmission of cold stress is closely correlated to the core CBF (C-REPEAT BINDING FACTOR) regulatory pathway, which plays an important role in the plant cold stress response (Liu et al., 2018). To understand the potential mechanisms of altered cold tolerance in *CsWAK12* transgenic plants, we examined the relative expression of *CsWAK12* and *AtCBFs* (*AtCBF1*, *AtCBF2*, and *AtCBF3*) genes in WT and transgenic *Arabidopsis* under −6°C treatment with different duration by qRT-PCR and the normal condition as control. In general, *AtCBFs* in transgenic lines were suppressed before the cold treatment and rapidly induced by cold at 1.5 h. It was noteworthy that *AtCBF2* was significantly inhibited in transgenic lines at 3 h, while the differences in performance of *AtCBF1* and *AtCBF3* were insignificant between transgenic lines and WT. After 6 h, the expression of *AtCBFs* tended to be consistent eventually (**Supplementary Table S4B**). Owing to the expression changes of *AtCBFs*, we detected the relative expression of *AtICE1*, which was considered as the core transcriptional regulatory element of *AtCBFs*. It was not surprising that the expression level of *AtICE1* was stable and the differences between transgenic lines and WT were relatively subtle (**Figure 4**). Additionally, we examined several key cold response genes associated with *AtCBFs*, including *AtKIN1*, *AtCOR15A*, *AtCOR15B*, *AtCOR47*, and *AtRD29A*, using qRT-PCR (**Supplementary Figure 1**). These genes displayed distinct expression patterns, indicating that CsWAK12 may be involved in complex cold response mechanisms. For instance, the fluctuations in *AtCOR15A* expression were minimal in the WT, whereas they were significantly elevated in the transgenic lines. Furthermore, the expression of *AtCOR15B* in the transgenic line OE14 was higher than that of the WT under normal conditions, but decreased to levels comparable to the WT under cold treatment.

### 3.6 Transcriptome analysis of *CsWAK12* transgenic *Arabidopsis*

To investigate the potential molecular roles of *CsWAK12* in cold stress responses, we conducted a comparative transcriptome analysis of both WT and OE14 under normal growth conditions (N) and cold stress (C) using the Illumina Hiseq X Ten sequencing platform. A total of 99.33 GB raw reads were obtained from all tested samples. More than 87.86 GB average clean data ratio was above 95% (Q20 > 97.64% and Q30 > 93.73%) (**Supplementary Table S5**).

**Figure 5:**
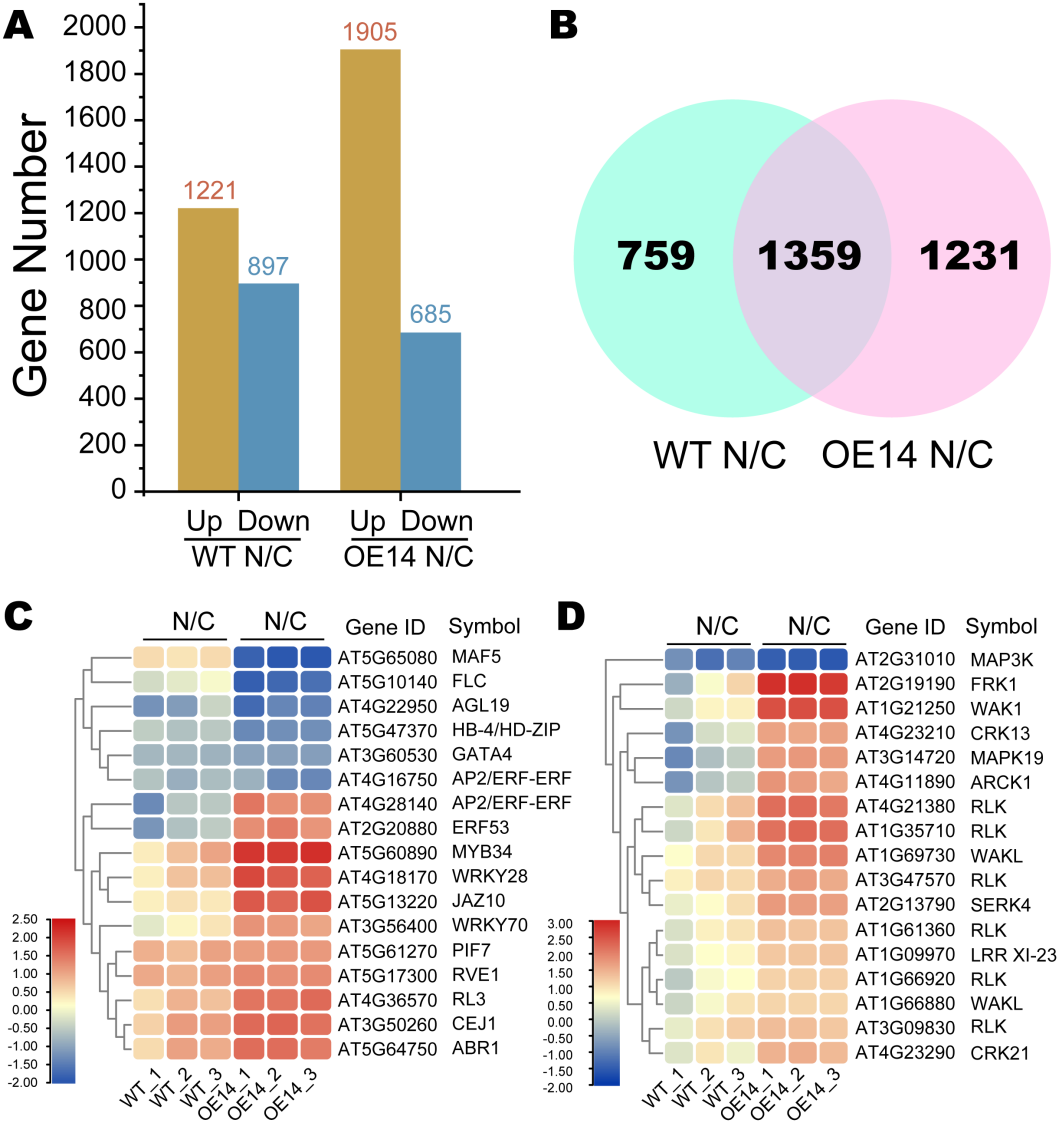
Classification of the assembled transcripts and the differentially expressed genes (DEGs). **(A)** Histogram of DEGs in two comparison groups between WT and OE14. **(B)** Venn diagram of OE14-specific DEGs in two comparison groups between WT and OE14. **(C)** Heatmaps of OE14-specific DEGs classified as TF. **(D)** Heatmaps of DEGs classified as PK. Log_2_(TPM Fold Change) ≥ 1 or ≤−1 and *p* < 0.05 were used as the standard for screening DEGs. The symbol “N/C” refers to a comparative transcriptome analysis of *Arabidopsis* plants under normal growth conditions (N) and cold stress **(C)**.

DEGs were analyzed between each sequential stage (WT N/C and OE14 N/C) based on fragments per kilobase of transcript per million mapped reads (FPKM), applying thresholds of false discovery rate (FDR) < 0.01 and FC > 2. In WT, we identified 1,221 upregulated and 897 downregulated genes, while in OE14, there were 1,905 upregulated and 685 downregulated genes (**Figure 5A**). Overall, a total of 3,349 DEGs were identified between the two comparison groups, with 759 DEGs specific to WT and 1,231 DEGs specific to OE14 (**Figure 5B**). To further clarify the function categories of the DEGs induced by cold stress, we conducted a GO and Kyoto Encyclopedia of Genes and Genomes (KEGG) enrichment analysis. Generally, a corrected *p*-value (*Q*-value) of less than 0.05 indicates significant enrichment of functions. As presented in **Supplementary Table S6**, the GO terms associated with DEGs in OE14 N/C significantly differ from those in WT N/C. Notably, the biological regulation of biological processes (GO:0065007, *Q*-value = 1.89E-14), the extracellular region of cellular components (GO:0005576, *Q*-value = 5.13E-12), and the catalytic activity of molecular functions (GO:0003824, *Q*-value = 7.54E-05) were particularly prominent. Furthermore, KEGG enrichment analysis of DEGs in OE14 N/C indicated that CsWAK12 may be involved in secondary metabolic pathways such as phenylpropanoid biosynthesis (ko00940, *Q*-value = 1.42E-05), alpha-linolenic acid metabolism (ko00592, *Q*-value = 5.40E-05), and glucosinolate biosynthesis (ko00966, *Q*-value = 6.63E-03) (**Supplementary Table S7**). These results suggest that the cold response mechanisms regulated by CsWAK12 in plants are complex.

The specific DEGs in OE14 N/C were predicted using iTAK, revealing 17 transcription factors (TFs) and 17 protein kinases (PKs) that exhibited significant expression changes under cold stress (**Figures 5C, D**). The expression of TFs such as MAF5 (AT5G65080) and FLC (AT5G10140) was significantly inhibited under cold stress, whereas MYB34 (AT2G31010), WRKY28 (AT4G18170), and JAZ10 (AT5G13220) were significantly activated (**Supplementary Table S8A**). Notably, ERF (AT4G28140) expression was reduced in WT under cold stress but was highly induced in the overexpressing (OE) line. Regarding PKs, genes such as MAPK3 (AT2G31010) were significantly inhibited under cold stress, while FRK1 (AT2G19190) and WAK1 (AT1G21250) were significantly activated (**Supplementary Table S8B**).

The genes involved in the CBF pathway contribute to the formation of cold resistance in plants (**Supplementary Table S10**) (Kidokoro et al., 2021, 2023). To investigate the underlying mechanisms of cold sensitivity in transgenic plants, we analyzed the expression patterns of 40 genes associated with the CBF pathway using transcriptome data (**Supplementary Table S11**). The result showed that the expression of circadian clock-related genes, such as *REVEILLE 4* (*RVE4*), *Central Circadian Oscillator 1* (*CCA1*), *Late Elongate Hypocotyl* (*LHY*), and *Night Light-inducible and Clock-Regulated* (*LNKs*), was significantly induced in the OE line. In contrast, several TF genes, including *ERF53/54*, *CBFs*, and *DREB2A*, were markedly reduced in the OE line (**Supplementary Figure 3**). These findings suggest that CsWAK12 may play a role in the biological processes related to plant biorhythms, thereby influencing plant cold tolerance by inhibiting the expression of positive regulatory TFs.

## 4 Discussion

Multiple RLKs have been confirmed to play a role in the response to cold stress. CRPK1 (Cold-responsive protein kinase 1) negatively regulates cold tolerance by phosphorylating 14-3-3λ, which has been shown to promote the destabilization of CBF proteins via the 26S proteasome pathway in *Arabidopsis* (Liu et al., 2017). Overexpression of *MRLK2* (FERONIA gene) in apple enhances the expression of the cold-responsive genes and anthocyanin biosynthesis-related genes, resulting in increased cold tolerance in the OE lines (Jing et al., 2023). Moreover, overexpressing *BRI1* in *Arabidopsis* facilitates seed germination under cold stratification, whereas the *bri1-5* mutant exhibits significantly reduced sensitivity to cold stratification and inhibits the expression of CBF1/2 genes (Kim et al., 2019). It is intriguing to consider whether the WAK gene is also involved in cold response functionality. In this study, we report that a cell wall-associated RLK from tea plant, *CsWAK12*, serves as a potential negative regulator of the cold stress response. *CsWAK12* is induced by cold in the tea plant and reduces cold tolerance in *Arabidopsis* lines overexpressing *CsWAK12*, as evidenced by decreased seedling survival rates, SOD activity, and MDA content.

WAKs are considered as physical connections between the cell walls and the plasma membrane in higher plant. Gold conjugated secondary antibodies targeting the WAK1 antiserum localize to the surfaces of *Arabidopsis* leaf cells, particularly in the cell wall region, which can be visualized using both light and electron microscopy (He et al., 1996). Green fluorescence was observed along the edge of the cells after the transformation of the GFP-tagged CaWAKL20 coding sequence from pepper into onion epidermal cells (Wang et al., 2019). Similarly, in the case of the tea plant, the fusion protein CsWAK12-GFP was localized to the cell membrane after being introduced into tobacco leaves in this study (**Supplementary Figure 2**).

WAK proteins have been shown to play a significant role in plant growth and development. Antisense expression of *WAK* in *Arabidopsis* leaves, which resulted in a 50% reduction in total WAK protein levels, led to smaller leaf cells and consequently to dwarf plants (Lally et al., 2001; Kohorn et al., 2006). In rice, various WAK genes regulate growth through distinct mechanisms. *OsWAK10* and its variants modulate cell wall signals to control the amplitude of secondary wall cellulose synthesis by amplifying pectin-derived signals, which, in turn, influences the height of rice stems (Cai et al., 2023). Conversely, OsWAK11 negatively regulates the stature, leaf angle, and seed length of rice plants by phosphorylating and repressing OsBRI1 activity, a crucial regulatory site for rice architecture (Yue et al., 2022). Therefore, WAKs may exhibit differential roles in plant growth and development. In our study, *CsWAK12* was found to promote growth in *Arabidopsis* OE lines, enhancing root length, seed size, and overall growth potential (**Figure 3**). It is acknowledged that plants may suppress growth to redirect energy toward defense against environmental stress. Regarding the specific function of the *CsWAK12* gene, it appears to inhibit the cold stress response while simultaneously promoting growth, indicating heightened sensitivity to cold. The intriguing functional model of *CsWAK12* in relation to cold stress and physiological growth warrants further elucidation.

To investigate the functional model in depth, we analyzed the relationship between *CsWAK12* and the classical cold response CBF pathway. CBFs play crucial roles in plant response to cold stress, as their expression is rapidly induced by cold conditions. This induction enables CBFs to bind to the promoter regions of Cold-Regulated (COR) genes, activating their expression and thereby positively regulating cold resistance in plants. In our research, we observed the expression of CBF genes in WT and OE lines during cold treatment. Notably, the expression of *CBFs* in OE lines was suppressed at 1.5 h but induced at 3 h under normal conditions. Although cold stress induced *CBF* expression, we noted considerable variation in the magnitude of these changes between WT and OE lines. These results suggest that CsWAK12 may be involved in the CBF pathway, potentially reducing cold resistance by interfering with the transcriptional activity of *CBFs*. Inducer of CBF expression 1 (ICE1) is a master regulator of CBFs, encoding an MYC-like bHLH TF, that binds to canonical MYC cis-elements in *CBF* promoters, thereby positively regulating *CBF* gene expression (Miura et al., 2007). As a core component of the CBF signaling pathway, the transcriptional level of *ICE1* is not induced by low temperature; however, its protein level is regulated by various modifications, including ubiquitination and phosphorylation (Ye et al., 2019). In our study, the relative expression of *ICE1* was only slightly affected by low temperature in both WT and transgenic lines, consistent with previous findings. We speculate that *CsWAK12* may play a role in the post-translational modification of ICE1, which subsequently influences the expression of *CBFs.*

Numerous reliable studies have confirmed that TFs such as MYB, WRKY, ERF, and JAZ are involved in the cold stress response in plant (Xu et al., 2018; Li et al., 2023; An et al., 2022). MdMYB308L interacts with MdbHLH33, enhancing its binding to the promoters of *MdCBF2* and *MdDFR* in apple (Xie et al., 2018). *Arabidopsis* plants overexpressing PbMYB1L from pear exhibited significant anthocyanin accumulation in leaves following cold treatment, which significantly induced the expression of *AtCBF* genes, resulting in enhanced cold tolerance (Zhou et al., 2024). WRKY33 in both wild and cultivated tomatoes positively regulates cold tolerance (Guo et al., 2022). In rice, WRKY53 mediates the crosstalk between brassinosteroid (BR) signaling and the MAPK pathway to regulate plant architecture and seed size, while negatively regulating rice cold tolerance at the booting stage by fine-tuning anther gibberellin levels (Tang et al., 2022; Tian et al., 2021). Our previous research found that transgenic *Arabidopsis* lines overexpressing CsWRKY29 and CsWRKY37 genes exhibited higher survival rates and lower MDA levels under freezing treatment compared to WT plants (Zhao et al., 2022). In this study, we also observed that *CsWAK12* altered the expression of several TF genes. MYB34, WRKY28, and WRKY70 were inhibited in transgenic lines under normal conditions but were more strongly induced by low temperatures (**Supplementary Table S5A**). These results suggest that CsWAK12 plays a negative role in activating cold response TF genes. Therefore, it would be interesting to investigate the downstream cooperators of CsWAK12 to elucidate the cold response pathway.

Mitogen-activated protein kinases (MAPKs) represent a crucial signaling link between cell surface receptors and both transcriptional and enzymatic regulation in eukaryotes. Evidence suggests a role for MAPKs in WAK signaling. Protoplasts from plants homozygous for the null allele wak2-1 exhibited a reduction in the activation of MPK3 (Kohorn et al., 2009). MPK6 is likely a target of WAK2cTAP, a dominant allele of WAK2, which serves a distinct function compared to MPK3 (Kohorn et al., 2012). Our research demonstrated that MAP3K was highly induced in the OE line, while MAPK19 was inhibited under normal growth conditions. However, MAPK19 was induced by cold in the OE line. These results indicate a role for MAPKs in CsWAK12 signaling during cold stress. Furthermore, the expression of *WAK1* in the OE line was initially inhibited but was subsequently strongly induced by cold, whereas *CsWAK12* exhibited the opposite pattern. This suggests that CsWAK12 has an antagonistic relationship with WAK1 in response to cold. Future studies will be intriguing to explore the potential complementary functions of the *CsWAK12* and *WAK1* genes.

## 5 Conclusion

In this study, we report that tea CsWAK12 possesses conserved domains characteristic of the WAK family and is localized to the plasma membrane. The expression of CsWAK12 is upregulated in response to cold stress. Overexpression of CsWAK12 promotes plant growth but decreases *Arabidopsis* tolerance to cold stress. Additionally, overexpressing CsWAK12 reduces the cold-induced expression of CBF genes under normal conditions, although their expression is more strongly induced by cold stress. Therefore, we suggest that CsWAK12 negatively modulates plant cold tolerance by interfering with the transcriptional activity of *CBFs*. Our results lay a foundation for further understanding the functional mechanisms of WAKs in plant adaptation to environmental stress.

## Data Availability

The RNA-seq raw data for all samples are accessible at NCBI BioProject PRJNA1102880, which includes an accession number for SRX24324921~SRX24324932. The isolated full-length mRNA of CsWAK12 is available at NCBI Nucleotide Datasets under the accession number PP739783. The original contributions presented in the study are included in the article/**Supplementary Material**, further inquiries can be directed to the corresponding author/s.
