# Supplementary material for: CsWAK12, a novel cell wall-associated receptor kinase gene from Camellia sinensis, promotes growth but reduces cold tolerance in Arabidopsis
Source: Front Plant Sci. 2024 Nov 28;15:1420431. doi: 10.3389/fpls.2024.1420431 (PMC11634587; doi:10.3389/fpls.2024.1420431)
Supplement: Supplementary file 1 [file DataSheet1.pdf]

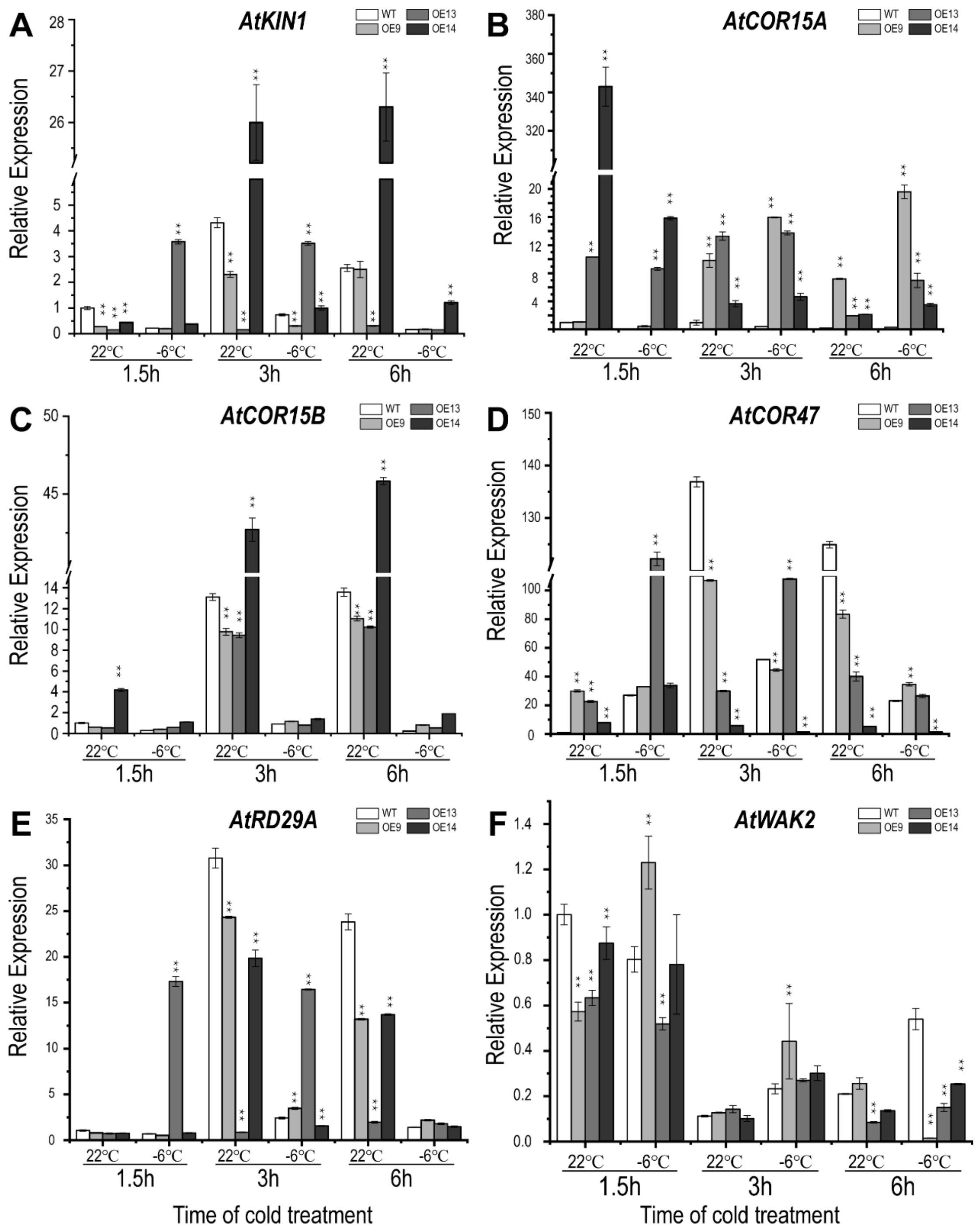

Supplementary Figure 1 Expression pattern of cold-related genes in Arabidopsis plants (A) The expression of *AtKIN1* (B) and *AtCOR15A* (C) and *AtCOR15B* (D) and *AtCOR47* (E) and *AtRD29A* (F) and *AtWAK2* in transgenic lines and WT. The error bars indicate the SDs from three biological replicates. ** indicates a significant difference compared with WT under each treatment at the 0.01 level.
