# Supplementary material for: CsWAK12, a novel cell wall-associated receptor kinase gene from Camellia sinensis, promotes growth but reduces cold tolerance in Arabidopsis
Source: Front Plant Sci. 2024 Nov 28;15:1420431. doi: 10.3389/fpls.2024.1420431 (PMC11634587; doi:10.3389/fpls.2024.1420431)
Supplement: Supplementary file 2 [file DataSheet2.pdf]

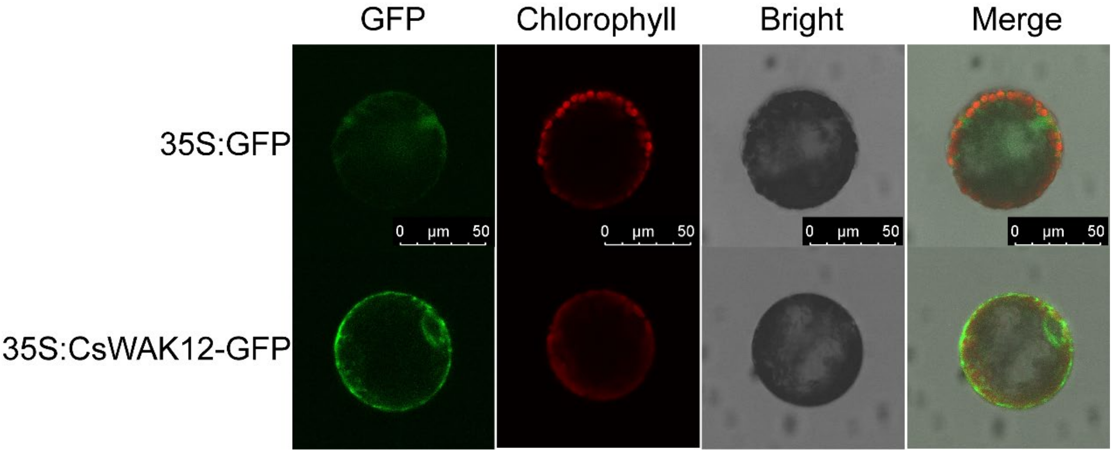

Supplementary Figure 2 Subcellular localization CsWAK12 protein

Green fluorescence was exclusively observed in the membrane of cells expressing CsWAK12, while the GFP signal from the control was detected in the cytoplasm and nucleus.
