# Supplementary material for: CsWAK12, a novel cell wall-associated receptor kinase gene from Camellia sinensis, promotes growth but reduces cold tolerance in Arabidopsis
Source: Front Plant Sci. 2024 Nov 28;15:1420431. doi: 10.3389/fpls.2024.1420431 (PMC11634587; doi:10.3389/fpls.2024.1420431)
Supplement: Supplementary file 3 [file DataSheet3.pdf]

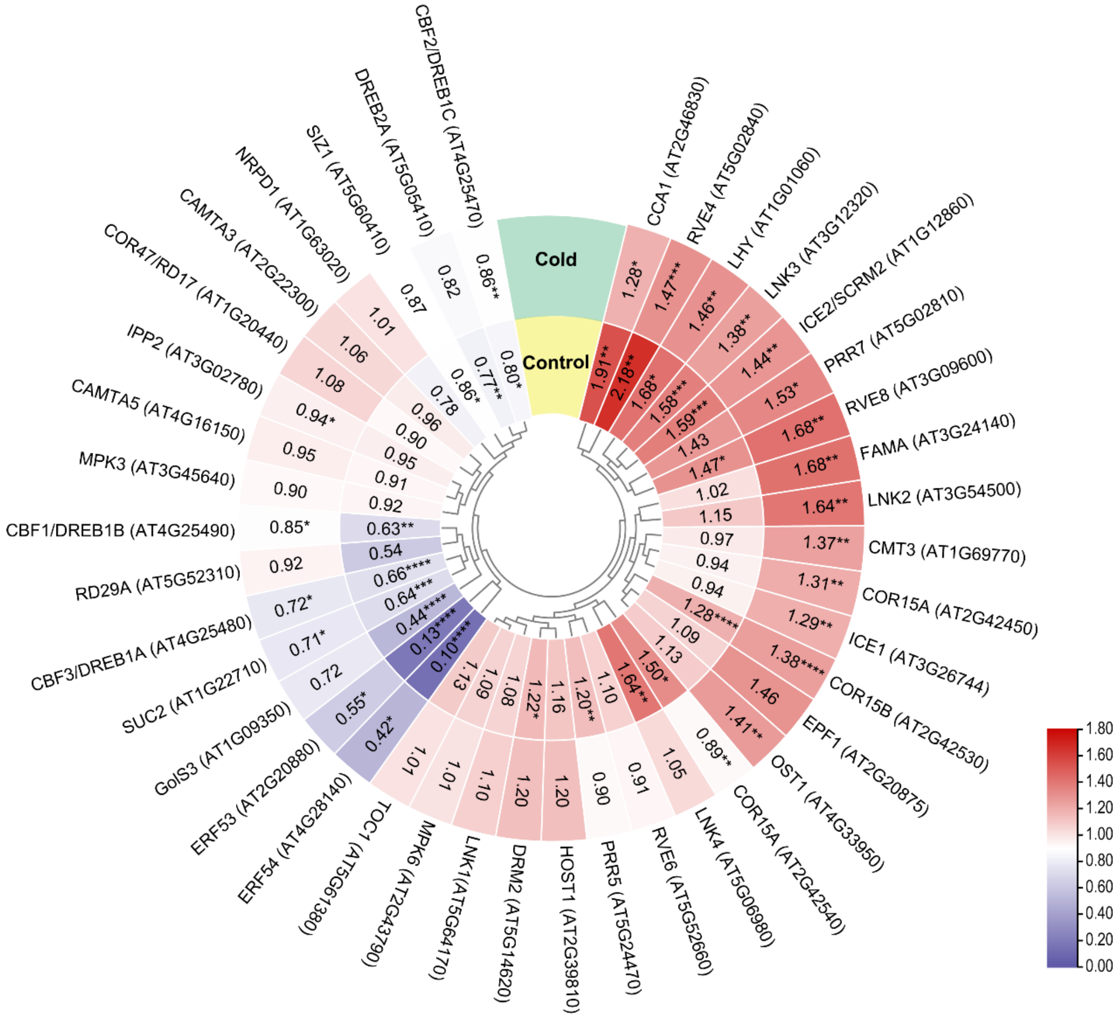

Supplementary Figure 3 The differential gene expression of the CBF pathway genes identified in OE and WT under cold treatment

The heatmap in the outer circle illustrates the log2 fold changes observed in response to cold conditions (4°C), while the inner circle depicts the log2 fold changes under normal control conditions for OE14 compared to WT. The numbers within the color blocks represent the actual multiples. Statistically significant differences were indicated by: *, $p < 0.05$; **, $p < 0.01$; ***, $p < 0.001$; ****, $p < 0.0001$ according to unpaired t-test.
